# Plasma pharmacokinetic profile of fluralaner (Bravecto™) and ivermectin following concurrent administration to dogs

**DOI:** 10.1186/s13071-015-1123-8

**Published:** 2015-10-06

**Authors:** Feli M. Walther, Mark J. Allan, Rainer KA Roepke

**Affiliations:** Merck Animal Health, 2 Giralda Farms, Madison, NJ USA; MSD Animal Health Innovation GmbH, Zur Propstei, 55270 Schwabenheim, Germany

**Keywords:** Fluralaner, Bravecto™, Ivermectin, Dog, Pharmacokinetic, P-glycoprotein, MDR1

## Abstract

**Background:**

Fluralaner is a novel systemic ectoparasiticide for dogs providing immediate and persistent flea, tick and mite control after a single oral dose. Ivermectin has been used in dogs for heartworm prevention and at off label doses for mite and worm infestations. Ivermectin pharmacokinetics can be influenced by substances affecting the p-glycoprotein transporter, potentially increasing the risk of ivermectin neurotoxicity. This study investigated ivermectin blood plasma pharmacokinetics following concurrent administration with fluralaner.

**Findings:**

Ten Beagle dogs each received a single oral administration of either 56 mg fluralaner (Bravecto™), 0.3 mg ivermectin or 56 mg fluralaner plus 0.3 mg ivermectin/kg body weight. Blood plasma samples were collected at multiple post-treatment time points over a 12-week period for fluralaner and ivermectin plasma concentration analysis.

Ivermectin blood plasma concentration profile and pharmacokinetic parameters C_max_, t_max_, AUC_∞_ and t½ were similar in dogs administered ivermectin only and in dogs administered ivermectin concurrently with fluralaner, and the same was true for fluralaner pharmacokinetic parameters.

**Conclusions:**

Concurrent administration of fluralaner and ivermectin does not alter the pharmacokinetics of either compound. Based on the plasma pharmacokinetic profile and the clinical observations, there is no evident interaction between fluralaner and ivermectin, and co-administration does not increase the risk of ivermectin associated neurotoxicity.

## Findings

### Background

Fluralaner is a novel systemically administered isoxazoline class compound that provides immediate and persistent insecticidal and acaricidal efficacy after oral administration to dogs. A field study has shown that a single fluralaner dose administered orally to dogs provides at least 12 weeks of flea and tick control [1] and another study demonstrated efficacy against mites (*Demodex* spp.) [2]. Fluralaner was shown to be safe when administered orally at overdoses of up to 5 times the maximum clinical dose at 8-week intervals in healthy Beagle dogs [3] and at overdoses of 3 times the maximum clinical dose in Collies bearing a homozygous defect of the multi-drug-resistance 1 gene (MDR1 −/−) [4]. There are no known interactions of fluralaner with other veterinary medicinal drugs [5] and fluralaner was shown to be safe when administered concurrently with macrocyclic lactones like milbemycin oxime [6] and moxidectin [7].

Ivermectin is registered for the use in dogs at monthly oral doses of 6 mcg/kg BW for heartworm protection [8]; some veterinarians may choose to administer ivermectin at higher off label doses to treat dogs for different worm or mite infestations (for example 0.05 mg/kg for hookworm, 0.1 mg/kg BW for whipworms, 0.2 mg/kg for *Toxocara canis*, 0.2-0.4 mg/kg for sarcoptic mange, 0.2 mg/kg for nasal mites *Pneumonyssus caninum*, 0.3 mg/kg for cheyletiellosis, 0.3–0.6 mg/kg for demodicosis; orally or subcutaneous as single or repeated treatments) [9–20]; however, such high doses of ivermectin cannot safely be administered to “ivermectin-sensitive” dogs carrying a MDR1 mutation [21, 22]. Ivermectin is a substrate for the p-glycoprotein (p-gp) transporter encoded by the MDR1 gene [22, 23]. This transporter limits the entry of its substrates into the body by an efflux-based mechanism, particularly at the blood–brain barrier [24]. Dogs with a homozygous defect of the MDR1 gene do not carry a functional p-glycoprotein transporter and are therefore more susceptible to neurotoxicity caused by ivermectin [21]. Furthermore, drug-drug interactions at the p-glycoprotein transporter may occur following the concurrent use of ivermectin and drugs, leading to an increased risk of neurotoxicity of ivermectin in MDR1 intact dogs. One example is spinosad that inhibits the p-glycoprotein transporter-mediated elimination of ivermectin in MDR1 intact dogs, thereby increasing ivermectin blood concentrations, which leads to a higher risk of neurotoxicity when administering high off-label doses of ivermectin concurrently with spinosad [25–30].

Veterinarians may choose to administer fluralaner and ivermectin concurrently. To ensure that the concurrent use does not increase the risk of ivermectin-associated neurotoxicity, the pharmacokinetic profile of ivermectin was investigated when administered concurrently with fluralaner. For pharmacokinetic characterization over time, fluralaner and ivermectin were administered at high dose rates (i.e. 56 mg fluralaner/kg BW, the highest expected dose in clinical use, and 0.3 mg ivermectin/kg BW) and on a single occasion.

### Methods

Thirty healthy Beagle dogs (15 males and 15 females) were included in the study. Dogs were kept indoors in pens with sealed floors and were housed in groups of two or three, with the exception of the 3 days after ivermectin/fluralaner administration, when dogs were housed individually. Dogs had access to water *ad libitum* throughout the study period and were fed a standard dog diet.

This study was conducted in Ireland in compliance with Directive 2010/63/EU S.I. No. 543 of 2012 and the Irish national animal protection legislation framework (experimental license no. B100\4500), and the study plan was approved by the research organization institutional (Charles River Laboratories Preclinical Services Ireland Ltd.) ethics committee.

The 30 dogs were allocated to three study groups by sorting within gender according to descending body weight and random allocation to a group (Table 1). Ivermectin (Ivomec Classic Injection for Cattle and Sheep; Merial Animal Health) was administered orally at a dose of 0.3 mg/kg BW and fluralaner (Bravecto™; Merck/MSD Animal Health) was administered orally at the maximum clinical dose of 56 mg/kg BW on study day 0. Blood samples for plasma concentration determination were collected prior to administration and at 1, 2, 4, 6, 8, 10, 24, 48, 72, 120, 168, 240, 336, 504, 672, 1008, 1344, 1656 and 2016 h (84 days) after administration. Ivermectin and fluralaner blood plasma concentrations were determined using validated methods (lower limit of quantification 1 ng ivermectin/mL and 10 ng fluralaner/mL).

**Table 1:** Study groups for evaluation of the pharmacokinetic profile of ivermectin and fluralaner when administered concurrently to dogs

		Ivermectin	Fluralaner	Ivermectin plus Fluralaner
Ivermectin dose (mg/kg BW)		0.3	-	0.3
Fluralaner dose (mg/kg BW)		-	56	56
Gender	Male	5	5	5
	Female	5	5	5
Body weight (kg)	Mean ± SD	13.1 ± 1.2	13.3 ± 1.5	13.1 ± 1.0

*SD* standard deviation

Standard pharmacokinetic parameters including maximum plasma concentration (C_max_), time to C_max_ (t_max_), the extrapolated area under the curve (AUC_∞_) and the elimination half-life (t½) were calculated using non-compartmental and linear trapezoidal methods. Statistical analysis of pharmacokinetic parameters was performed after natural logarithmic transformation, with the exception of t_max_, using ANOVA models and 90 % confidence intervals, and the individual animal being the experimental unit. Pharmacokinetic and statistical analyses were performed using SAS/STAT® (Language: Reference, Version 9.3, SAS Institute Inc., Cary, NC, USA).

### Results and discussion

**Fig. 1:**
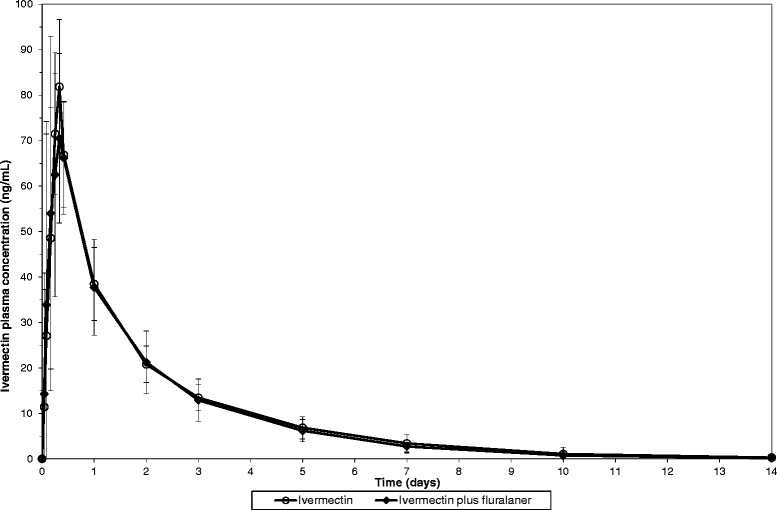
Mean ivermectin plasma concentration (± standard deviation) in dogs following oral administration (0.3 mg/kg BW) alone or concurrently with fluralaner (56 mg/kg BW)

**Fig. 2:**
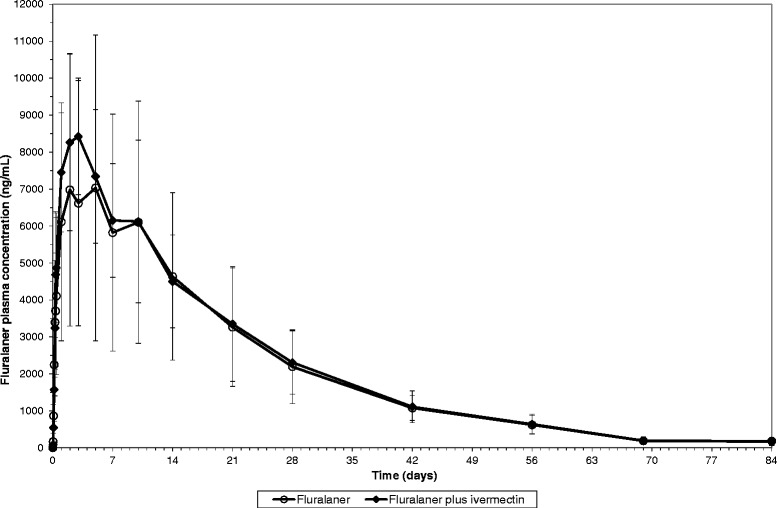
Mean fluralaner plasma concentration (± standard deviation) in dogs following oral administration (56 mg/kg BW) alone or concurrently with ivermectin (0.3 mg/kg BW)

The plasma concentration versus time profile of ivermectin was comparable in dogs administered ivermectin only and in dogs administered ivermectin concurrently with fluralaner (Fig. 1). Similarly, the plasma concentration versus time profile of fluralaner was comparable in dogs administered fluralaner only and in dogs administered fluralaner concurrently with ivermectin (Fig. 2). The pharmacokinetic parameters of both, ivermectin and fluralaner, were also comparable across groups (Tables 2 and 3), with no statistical significant differences between groups.

**Table 2:** Ivermectin pharmacokinetic parameters in dogs following oral administration (0.3 mg/kg BW) alone or concurrently with fluralaner (56 mg/kg BW)

Parameter	Unit	Ivermectin	Ivermectin plus Fluralaner	*P-*value
		Mean ± SD	Mean ± SD	
C_max_	(ng/mL)	92.70 ± 26.77	80.52 ± 21.41	0.2465
t_max_	(day)	0.29 ± 0.10	0.31 ± 0.11	0.7269
AUC_∞_	(day*ng/mL)	141.96 ± 27.23	134.26 ± 37.99	0.5073
t½	(days)	2.07 ± 0.71	1.84 ± 0.42	0.4888

*SD* standard deviation

**Table 3:** Fluralaner pharmacokinetic parameters in dogs following oral administration (56 mg/kg BW) alone or concurrently with ivermectin (0.3 mg/kg BW)

Parameter	Unit	Fluralaner	Fluralaner plus Ivermectin	*P-*value
		Mean ± SD	Mean ± SD	
C_max_	(ng/mL)	7976 ± 4239	9312 ± 1767	0.1529
t_max_	(day)	3.00 ± 1.49	3.20 ± 2.66	0.8379
AUC_∞_	(day*ng/mL)	175778 ± 75122	184030 ± 49524	0.5373
t½	(days)	14.27 ± 2.53	13.45 ± 1.68	0.5107

*SD* standard deviation

### Conclusions

Concurrent administration of fluralaner and ivermectin does not alter the pharmacokinetics of either compound. There is no evident interaction of fluralaner and ivermectin indicating an increased risk of ivermectin-associated neurotoxicity in fluralaner-treated dogs.
